# High prevalence of malaria in a non-endemic setting among febrile episodes in travellers and migrants coming from endemic areas: a retrospective analysis of a 2013–2018 cohort

**DOI:** 10.1186/s12936-021-03984-9

**Published:** 2021-11-27

**Authors:** Alejandro Garcia-Ruiz de Morales, Covadonga Morcate, Elena Isaba-Ares, Ramon Perez-Tanoira, Jose A. Perez-Molina

**Affiliations:** 1 Infectious Diseases Department, National Referral Centre for Tropical Diseases, Hospital Universitario Ramón y Cajal, Instituto Ramón y Cajal de Investigación Sanitaria (IRYCIS), CIBER de Enfermedades Infecciosas, 28034 Madrid, Spain; 2 Internal Medicine Department, Móstoles University Hospital, Móstoles, Spain; 3 Clinical Microbiology Department, Príncipe de Asturias University Hospital, Madrid, Spain; 4 Biomedicine and Biotechnology Department, Faculty of Medicine, Alcalá de Henares University, Alcalá de Henares, Spain

**Keywords:** Fever, Diagnosis, Malaria, Travellers, Migrants, Spain, VFR, Positive rate, Quality control

## Abstract

**Background:**

The study aimed to analyse the likelihood of imported malaria in people with a suggestive clinical picture and its distinctive characteristics in a hospital in the south of Madrid, Spain.

**Methods:**

Observational retrospective study that consisted of a review of all medical files of patients with any malaria test registered at Móstoles University Hospital between April 2013 and April 2018. All suspected malaria cases were confirmed by *Plasmodium* spp. polymerase chain reaction (PCR).

**Results:**

Of the 328 patients with suspected malaria (53.7% migrant-travellers; 38.7% visitors; 7.6% travellers), 108 cases were confirmed (101 by *Plasmodium falciparum*), accounting for a 33% positive sample rate. Sixteen cases were diagnosed only by PCR. Patients with malaria, compared to those without, presented predominantly with fever (84% vs. 65%), were older (34 vs. 24 years), sought medical attention earlier (17d vs. 32d), had a greater number of previous malaria episodes (74% vs. 60%), lower levels of platelets (110,500µL vs. 250,000µL), and higher of bilirubin (0.6 mg/dL vs. 0.5 mg/dL). Severe malaria was present in 13 cases; no deaths were recorded. Malaria diagnosis showed a bimodal distribution with two peaks: June to September and November to January.

**Conclusions:**

Malaria is still a common diagnosis among febrile patients coming from the tropics specially among migrant travellers. Fever, thrombocytopenia, and/or high bilirubin levels should raise suspicion for this parasitic infection. Prompt diagnosis is crucial to avoid severe cases and deaths.

**Supplementary Information:**

The online version contains supplementary material available at 10.1186/s12936-021-03984-9.

## Background

Malaria is not only the most prevalent and lethal vector-borne disease, but also one of the most relevant imported parasitic infections worldwide. Because of its potentially lethal complications, it is considered a leading public health concern by the World Health Organization (WHO) [1]. Malaria is endemic in 91 countries, and nearly half of the world’s population is at risk. According to WHO estimates, the global burden of malaria in 2018 was 228 million cases, most (93%) originating from the WHO African Region, with an estimated incidence rate of 57 cases per 1000 at-risk persons. Deaths in 2017 were estimated at 405,000, 94% of which occurred with-in the WHO African Region [1].

Since malaria was declared eradicated from Europe in 1978, cases have largely been imported by international travellers and migrants from endemic regions. According to the European Centre for Disease Control (ECDC), 8349 cases were reported in 2018, with France, UK, Germany, and Spain accounting for more than 50% of cases. Data suggest that due to growing immigration rates towards Europe, a large proportion of imported malaria cases occur among recent immigrants from malaria-endemic countries or among settled migrants and their families who travel to malaria-endemic home countries [2, 3]. Additionally, a growing number of travellers visit malaria-endemic areas each year [4].

Spain has 46.7 million inhabitants [5], 10% of whom are immigrants, and it is considered Europe’s port of entry from the WHO African region. Despite its history as a malaria-endemic country, Spain was declared malaria-free in 1964, with the last autochthonous case reported in 1961 [6]. Since its purported eradication, more than 10,000 cases have been reported. Due to changing migration patterns and travelling habits the last 10 years account for more than half of them, with an average of 600 cases per year [7], nearly all considered imported. Hence, cases of locally acquired malaria have been suspected during the past decade in situations where a history of travel to an endemic zone was absent; however, local sources of infection could not be identified. Given the presence of some Anopheline species (*Anopheles atroparvus*) in a variety of regions, the possibility of a new outbreak of autochthonous cases in the near future cannot be excluded [6].

As with all other European countries, most malaria cases in Spain affect immigrants and their offspring who return to their home countries to visit friends and relatives (VFR). VFR represent a special risk population [8, 9] as a result of, for example last-minute travel, lack of pretravel consultation, absence of malaria chemoprophylaxis and longer stays in local communities, where there is a higher transmission risk, among other factors [9–12].

According to a multicentre study from the GeoSentinel surveillance network, malaria was the most frequent cause of fever (29%), followed by dengue, typhoid fever, chikungunya and rickettsiosis [13]. Further, malaria was the first cause of infection-related death among travellers, accounting for nearly 25% of cases [14]. Delays in diagnosis are the main determinant for a higher morbidity and risk of mortality [15]. Correctly differentiating malaria cases from other febrile conditions must be prioritized in patients returning from a malaria-endemic country [13].

This study aimed to analyse the likelihood of malaria in individuals presenting with a suggestive clinical picture and to distinguish the characteristics of malaria patients from those without malaria.

## Methods

### Study population and study design

A retrospective, descriptive, analytic study was carried out using the medical files of all patients with any malaria test registered at Móstoles University Hospital, between April 2013 and April 2018. Due to the 24-hour availability of trained microscopist, no rapid diagnostic tests (RDTs) are performed at the hospital, and all thin or thick smears are automatically followed by real-time polymerase chain reaction (RT-PCR) to confirm the results, even in cases where parasites are not detected. Móstoles University Hospital is a second level hospital in the region of Madrid, with 6.6 million inhabitants and a 13.4% registered immigrant population (893,276); by country, Romanians represent the biggest number (21.7%), followed by Moroccans (8.8%); whereas according to geographic region, Central and South Americans represent 32.3%, and sub-Saharan African migrants 3.8% [16].

A data extraction sheet was prepared that gathered demographic data such as age, gender, country of birth, and country of permanent residence; travel-related data such as country of travel, previous visits to malaria-endemic countries, reason for the visit, time spent in an endemic country, and time since returning from an endemic country; disease-related data such as reason for consultation, symptoms (including fever), time since symptom onset, previous medical conditions, malaria chemoprophylaxis, *Plasmodium* species, need for admission or ICU admission, days of admission, and treatment administered; and biological data including blood count, serum chemistry, chest X-ray, and final diagnosis if not diagnosed with malaria.

Patients were divided into three groups: (a) immigrants that permanently live in Spain (and their offspring) visiting friends and relatives (VFR); (b) travellers to endemic areas; and (c) patients with permanent residence in an endemic country visiting Spain for any reason.

### Statistical analysis

In the descriptive study, continuous variables are described as mean and standard deviation (SD), and as median interquartile range (IQR) according to normality. Qualitative variables are expressed as percentages, and quantitative data are expressed as the median and IQR. The chi-squared and Fisher’s exact tests were used, when appropriate, for the comparison of categorical variables, whereas continuous variables were compared using the student *t*-test when normally distributed, or the Mann–Whitney U test when not normally distributed. A *p*-value <0.05 was considered statistically significant. All data were analysed using IBM® SPSS® Statistics 25 and GraphPad® Prism® 7 software.

## Results

A total of 382 samples—each of which included a thin smear, a thick smear, and a *Plasmodium* RT-PCR—were initially selected from patients with suspected malaria. Fifty-four patients were excluded from the study; 46 were follow-up tests in patients previously diagnosed with malaria, seven corresponded to newborns from malaria-infected mothers, and one was of nosocomial acquisition.

Thus, 328 samples corresponding to 328 patients were included in the study. Most patients were VFR (176; 53.7%), including 148 (45.1%) born in malaria endemic-countries (VFR-migrants) and 28 (8.5%) born in Spain (VFR-travellers). Furthermore, 127 (38.7%) patients were visitors and 25 (7.6%) were travellers from Spain (24) and Italy (1) to endemic countries. Most patients (215; 65.5%) originated from Equatorial Guinea, followed by 62 (18.9%) Spanish-born patients (including VFR travellers) and 38 (11.6%) Nigerian-born patients (Table 1). All three groups were similar, except that visitors were younger and travellers had had fewer previous malaria episodes.

**Table 1:** Main characteristics of the study population

Characteristics	Total (N = 328)	VFR (N = 176)	Travellers (N = 25)	Visitors (N = 127)
Region of travel or origin (for visitors)				
Sub-Saharan Africa	302	169	7	126
South and Central America	11	4	7	0
Southeast Asia and Indian subcontinent	9	0	8	1
Others/Unknown	6	3	3	0
Females, n (%)	188 (57%)	101 (57%)	9 (36%)	78 (61%)
Age (years), median (IQR)	30 (5-42)	33 (7-42)	35 (28-42)	19 (3-37)
Time elapsed since return from an endemic zone (days)	10 (5-20)	10 (5-21)	10 (7-15)	10 (4-22)
Travel to endemic country in the previous 5 years, n (%)	319 (97%)	169 (96%)	23 (92%)	127 (100%)
Previous malaria*	195/302* (65%)	99/158* (63%)	1/21* (5%)	95/123* (77%)
HIV+, n (%)	23 (7%)	9 (5%)	0 (0%)	14 (11%)
Hematologic disorders, n (%)**	17 (5%)	11 (6%)	1 (4%)	5 (4%)
Ongoing pregnancy, n (%)	12 (4%)	4 (2%)	0 (0%)	8 (6%)
Malaria +, n (%)	108 (33%)	68 (39%)	2 (8%)	38 (30%)

Unless otherwise stated, data is presented as n/N (%), where N is the group size for which the variable was documented

* 26 patients had no recorded data from previous malaria episodes, and were excluded from this analysis

** Hematologic disorders included: 12 sickle cell anemia, three cases of alfa thalassemia and two cases of sickle cell trait

A total of 89 thin smears and 90 thick smears identified malaria parasites (in 80 cases, both samples were positive), with six false positives among thin smears and none among thick smears. Sixteen cases of submicroscopic malaria were detected (negative thin and thick smears, but positive PCR), seven among VFR-migrants, three among VFR-travellers and six among visitors. Overall, 108 cases of malaria were confirmed by PCR representing a 33% among all suspected cases. In this study, thin smears showed 76% sensitivity and 97% specificity, whereas thick smears showed 83% sensitivity and 100% specificity.

Patients with malaria were older (34 vs. 24 years), took less time from return to seeking medical attention (17.6 vs. 32.5 days), and had suffered malaria more frequently in the past (74% vs. 55%; Table 2). Regarding clinical picture, fever was significantly more commonly cited as the reason for consultation among patients who were finally diagnosed with malaria (84% vs. 65%), while lower levels of platelets (110,500/µL vs. 250,000/µL) and increased bilirubin (0.6 mg/dL vs. 0.5 mg/dL) were the most characteristic analytical data.

**Table 2:** Comparison of clinical and biological findings in patients with and without malaria

	Total		*P*-value	VFR		Travellers		Visitors	
	Malaria + N = 108	Malaria – N = 220		Malaria + N = 69	Malaria – N = 107	Malaria + N = 2 ^a^	Malaria – N = 23	Malaria + N = 37	Malaria – N = 90
Age	34 (18–43)	24 (3–40)	<0.001	36 (22–44)	26 (3–41)	30, 53	35 (27–42)	29 (14–42)	11 (1–34)
Females	68 (63%)	120 (54.5%)	0.14	41 (59%)	60 (18%)	1 (50%)	8 (35%)	26 (70%)	52 (58%)
Time elapsed since return from an endemic zone (days) ^b^	17.6 (32)	32.5 (78.5)	0.019	16.7 (31.2)	30.3 (75.1)	13, 25	50 (104.3)	19.4 (35.1)	31.7 (76.8)
Days with fever	4 (2–6)	3 (1–6)	0.045	4 (2–6)	3 (2–7)	10, 15	3 (1–4)	3 (1–6)	2 (1–7)
Previous malaria^c^	74/100 (74%)	121/202 (60%)	0.016	44/63 (70%)	55/95 (58%)	0/2 (0%)	1/19 (5%)	30/35 (86%)	65/88 (74%)
Reason for consultation									
Fever	91 (84%)	142 (65%)	<0.0001	61 (88%)	69 (64%)	2 (100%)	16 (70%)	28 (76%)	57 (63%)
Bad general state	8 (7%)	17 (8%)	1	4 (6%)	10 (9%)	0 (0%)	3 (13%)	4 (11%)	4 (4%)
Digestive symptoms	2 (2%)	16 (7%)	0.06	0 (0%)	9 (8%)	0 (0%)	2 (9%)	2 (5%)	5 (5%)
Others	7 (6%)	45 (20%)	0.011	4 (6%)	19 (18%)	0 (0%)	2 (9%)	3 (8%)	24 (35%)
Biological findings									
Leukocytes /mcl	4825 (3485–6667)	6815 (5132–10460)	0.047	4890 (3550–6700)	6850 (5140–10130)	5640, 6120	6340 (4900–8755)	4525 (3445–6355)	6970 (5235–11540)
Hemoglobin (g/dL)	12.4 (10.7–13.4)	12.3 (11–13.4)	0.19	12.9 (11.8–13.7)	12.4 (11–13.5)	10.5, 13	13.65 (12.7–14.9)	11.1 (9.6–12.35)	11.8 (11–12.65)
Platelets/µL	110,500 (69750–162500)	250,000 (181000–326700)	<0.0001	110,000 (70500–148500)	249,000 (166000–313000)	490,000, 570,000	222,250 (194250–265500)	125,000 (74250–199250)	270,000 (204500–363000)
Bilirubin (mg/dL)	0.6 (0.4–1.1)	0.5 (0.3–0.6)	0.025	0.6 (0.4–1)	0.5 (0.3–0.7)	0.2, 5	0.5 (0.3–0.5)	0.5 (0.4–1.2)	0.4 (0.3–0.575)
Glycaemia (mg/dL)	94 (82–106)	90 (83–101)	0.49	97 (88–115)	90 (83–102)	89, 99	89 (85–104)	87 (78–101)	89 (79–98)
Creatinine (mg/dL)	0.8 (0.6–0.9)	0.6 (0.4–0.9)	0.18	0.8 (0.7–1)	0.6 (0.4 –0.9)	0.4, 0.8	0.8 (0.63–1.08)	0.7 (0.6–0.85)	0.6 (0.4–0.88)

Unless otherwise stated, data is presented as median (IQR) and n/N (%), where N is the group size for which the variable was documented

^a^Values for both patients are stated; ^b^Mean (SD); ^c^26 patients had no recorded data from previous malaria episodes and were excluded from this analysis

Most cases were diagnosed in VFRs (69; 64%) and visitors from endemic areas (37; 34%) and were predominantly caused by *P. falciparum* (101 cases; 93.5%), followed by *Plasmodium ovale* (5 cases; 4.6%; Table 3). Previous cases of malaria were common in visitors (30; 81%) and VFRs (44; 64%). Thirteen patients (12%) were diagnosed with severe malaria (WHO criteria [1]); nine VFR–migrants, three visitors and one traveller. One out of two malaria cases among travellers was severe, while no severe cases were found in VFR-travellers. Seventy-nine patients (73%) were admitted for a median of five days (IQR 3–6), while the remaining 29 were treated as outpatients. No malaria-related deaths were recorded during the study period.

**Table 3:** Characteristics of patients with malaria

Malaria +, n (%)	Total	VFR	Travellers	Visitors
	(N = 108)	(N = 68)	(N = 2)	(N = 38)
Patients with previous malaria episodes^a^	74 (74%)	44 (70%)	0	30 (86%)
Current episode causative species				
*P. falciparum*	101(93.5%)	65 (96%)	1 (50%)	35 (92%)
*P. vivax*	1 (0.9%)	0	1 (50%)	0
*P. ovale*	5 (4.6%)	3 (4%)	0	2 (5%)
*P. ovale* + *P. malariae*	1 (1.8%)	0	0	1 (3%)
Parasitation Index (%), Median (IQR)	0.75% (0.01–2.15)	0,9% (0.001–2)	1.3% (no data from the other)	0.45% (<0.1–2.15)
Admission, n (%)	79 (73%)	51 (74%)	1 (50%)	27 (73%)
Those of which in ICU	3 (2.8%)	2 (3.1%)	1 (50%)	0 (0%)
Severe Malaria	13 (12%)	9 (14%)	1 (50%)	3 (8%)
Days admitted, median (IQR)	5 (3–6)	4 (3–6)	65	5 (3.75–6)

Unless otherwise stated, data are presented as median (IQR) and n/N (%), where N is the group size for which the variable was documented

^a^26 patients had no recorded data from previous malaria episodes, and were excluded from that analysis

Regarding the 16 cases of submicroscopic malaria detected, 8 of them had been attended at the Infectious diseases outpatient clinic, whilst the remaining 8 came from the Emergency room. 10 had consulted due to fever and the other 6 had other symptoms suggestive of malaria (bad general state, headaches, diarrhoea). In those who consulted due to fever, 5 of them had a clear alternate diagnosis for their complains, including one HIV primoinfection, two Influenza B infections, one traveller’s diarrhoea and a tuberculoma; in the remaining 6 that consulted for other reasons, one was diagnosed of primary syphilis, one of preeclampsia and the third one of an upper respiratory tract infection.

Given the retrospective design of the study, indication for chemoprophylaxis could not be assured. Amongst 296 patients with registered information for having taken prophylaxis, no cases of malaria were diagnosed in 18 patients that took it correctly, whereas 101 cases were found among the 278 that did not take it.

Atovaquone-proguanil was the preferred drug for treatment (87 patients; 80%; Additional file 1: Table S2), whereas treatment for severe cases was based on intravenous artesunate (7 patients) followed by artemether/lumefantrine or atovaquone-proguanil. Primaquine was used in seven patients with non-falciparum malaria.

**Fig. 1:**
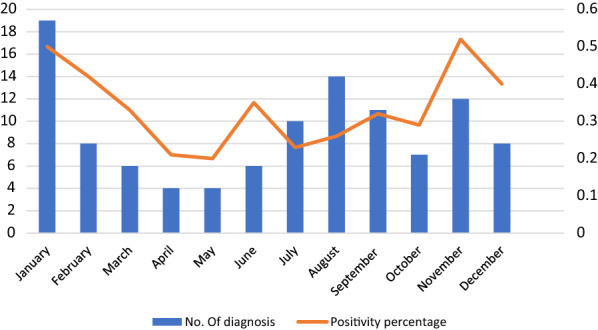
Monthly distribution of malaria diagnosis and percentage of positive samples during the study period (April 2013 to April 2018)

Regarding the number of malaria diagnoses, a bimodal temporal distribution was observed, with a first peak during Spanish summer (June to September) and a second peak from November to January. The sample’s positivity rate was higher from November to February, while the absolute number was higher from July to October (Fig. 1). There was no clear association between severe cases and any specific season: five cases (5/41; 12.2%) occurred from June to September and seven (7/47; 14.9%) from November to February (*p* = 0.71), while the remaining case occurred outside these periods.

Among the 220 patients suspected of malaria but ultimately having a different diagnosis, 79 (35.9%) suffered from upper respiratory tract infections, 20 (9.1%) from acute gastroenteritis or traveller’s diarrhoea, nine (4.1%) from tropical diseases not endemic to Spain, eight (3.5%) from active tuberculosis and two from primary HIV infection. The remaining 102 had a variety of 85 different diagnoses (Additional file 1: Table S3).

## Discussion

The aim was to describe the likelihood of malaria in patients with febrile episodes after returning from endemic regions in the south-west of Madrid. During the study period, 328 patients where malaria was suspected were evaluated, being one third of them (108) confirmed cases. Most severe cases were detected in VFR-migrants, no deaths were recorded, and only two cases (both travellers) could be considered late diagnoses because they sought care 10 and 15 days after symptom onset, respectively. In addition, the goal was to identify distinctive characteristics that could help diagnose malaria in such patients. In this sense, malaria was more frequent among migrants and VFRs, in those that had suffered previous episodes of malaria and who consulted due to fever; lower levels of platelets and increased total bilirubin were the most characteristic biological findings.

Fever is a common symptom among travellers returning from tropical areas [17], accounting for up to one-third of diagnoses upon return [9, 13] and causing up to 77% of hospital admissions [18]. Furthermore, among the potentially life-threatening tropical diseases acquired by travellers, the vast majority (malaria, typhoid fever, dengue, leptospirosis and melioidosis) present with it [14]. Hence, fever when returning from the tropics is a symptom that requires prompt evaluation, as it can indicate the onset of a severe illness. This is especially clear in malaria, where delays in diagnosis in imported cases is directly related to mortality [15]. Therefore, a high index of suspicion is essential, mainly when it does not imply high diagnostic costs (thin/thick smears and RDTs are low-cost exams).

Imported malaria is more commonly diagnosed in VFRs (migrants and travellers) and visitors, as compared to travellers [19, 20]. However, more severe cases are detected among travellers, followed by VFRs. Furthermore, visitors are found to have a lower risk for severe disease and ICU admission [15, 21–23]. Case fatality rates for imported malaria range from 0.06 to 1% in the UK, EU. and US [14, 15, 21–26], while figures for severe malaria range between 6% and 20%, being more common for *P. falciparum* malaria and in individuals with fewer previous episodes of malaria [14, 15, 27, 28]. The higher frequency of malaria diagnosis in VFRs and migrants is due to high-risk travel and greater exposure to malaria in their countries of origin [29–31]. Increased severity among travellers could be related to a lower index of suspicion and delayed diagnosis [15, 21], while the presence of some degree of malaria-immunity among migrants and VFRs would prevent them from presenting more severe cases [32]. Worst outcomes have also been described in children [29], mostly VFR-travellers with *P. falciparum* malaria acquired in sub-Saharan Africa, the elderly [15], and for cases detected in winter months, probably reflecting initial misdiagnosis of a febrile illness [15].

Imported malaria is more frequently diagnosed in male VFRs and visitors [20, 33]; nevertheless, this study showed a higher number of cases in females, which was probably due to the fact that most of the VFRs and visitors in our study came from Equatorial Guinea (126 VFRs, 119 visitors), who present a particular migration pattern to Spain, as compared to other African migrants, with higher percentage of females than males [5, 34].

In this way, 92% (303/328) of the diagnoses were made in VFRs and visitors, which is related to the high proportion of African migrants included in the study. Most cases were due to *P. falciparum*, with only one *Plasmodium vivax* case, as it would be expected due to their origin primarily in Western Africa (Additional file 1: Table S1). In this setting, the index of suspicion for malaria in the event of a febrile condition is high, probably influencing the absence of both mortality and a seasonal pattern for severe cases. It is noteworthy that one of the two travellers with malaria was a severe case; however because this patient only attended hospital 15 days after fever onset, he required immediate admission to ICU.

According to what is typically described [19, 20, 27, 35], differential clinical characteristics of a patient with malaria were fever, lower levels of platelets/leukocytes, and higher total bilirubin levels. Thus, fever should always be a warning sign in patients returning from a malaria-endemic area. Furthermore, laboratory results such as thrombocytopenia and higher levels of bilirubin are highly suggestive of *Plasmodium* spp. infection and, if absent, they have been proposed as biomarkers with negative predictive value [19, 20, 36].

Most cases were admitted into hospital even though IV treatment was not necessary for most of them. This event probably responds to the lack of possibilities for follow up by general practitioners, who are not used to treating malaria patients and the fact that a malaria test (smears or PCR) in Madrid can only be ordered from a hospital facility.

In 16 of the 108 cases (7.5%), *Plasmodium* spp. were only detected by PCR. All cases corresponded to VFR and visitors, none of whom developed severe malaria. A likely explanation is that the presence of a certain degree of immunity allowed better control of parasitaemia and lower concentrations of parasites [32] that were undetectable in smears. Submicroscopic parasitaemia also occurs in asymptomatic subjects, where it represents about 5% (4.5–5.7%) [37, 38] of screened migrants, even 28 months after returning from an endemic country. Persons with low-grade parasitaemia can infect mosquitoes [39], and thereby, could act as unidentified reservoirs and contribute to transmission in areas where malaria has been eradicated but that are still hosts to competent vectors [7].

The main strength of the study was the use of a systematic approach towards sick patients returning from malaria-endemic areas, and that every case in the cohort was analysed and confirmed by PCR. Thus, the population was well-characterized. However, it is a single-centre study with a very concrete migrant population (sub-Saharan Africa) and a high proportion of visitors and VFRs, so the results are not easily generalizable. These findings may not be applicable in settings where conventional travellers constitute the main population.

In conclusion, every fever with a travel history to an endemic region should be considered as malaria unless proven otherwise. The consequences of a delayed diagnosis can be fatal. This parasitosis should especially be presumed in patients with fever, low platelet levels, and raised levels of serum bilirubin.

## Supplementary Information

**Additional file 1.** Additional tables.

## Data Availability

Dataset of the study is available from the corresponding author upon reasonable request. CRediT authorship contribution statement. Jose A. Perez-Molina: Conceptualization, Writing - original draft, Writing - review & editing. Alejandro García-Ruiz de Morales: Conceptualization, data collection, Writing - original draft, Writing - review & editing. Covadonga Morcate: Data collection, Writing - review & editing. Elena Isaba: Data collection, Writing - review & editing. Ramón Pérez-Tanoira: Conceptualization, Writing - original draft, Writing - review & editing.
