# Supplementary material for: High prevalence of malaria in a non-endemic setting among febrile episodes in travellers and migrants coming from endemic areas: a retrospective analysis of a 2013–2018 cohort
Source: Malar J. 2021 Nov 27;20:449. doi: 10.1186/s12936-021-03984-9 (PMC8627073; doi:10.1186/s12936-021-03984-9)
Supplement: Supplementary file 1 — Additional file 1. Additional tables. [file 12936_2021_3984_MOESM1_ESM.docx]

**SUPPLEMENTARY MATERIAL**

Table S1. Geographical destination for travellers and VFRs and country of origin for visitors.

| **Country of travel** | Total  (N = 328) | VFR  (N = 176) | Travellers  (N = 25) | Visitors  (N = 127) |
| --- | --- | --- | --- | --- |
| Africa | 302 | 169 | 7 | 126 |
| Equatorial Guinea | 245 | 123 | 3 | 119 |
| Nigeria | 39 | 37 |  | 2 |
| Senegal | 5 | 2 |  | 3 |
| Mali | 4 | 4 |  |  |
| Cameroon | 3 | 3 |  |  |
| Tanzania | 1 |  | 1 |  |
| Mozambique | 2 |  | 2 |  |
| Burkina Faso | 1 |  | 1 |  |
| South Sudan | 1 |  |  | 1 |
| Zambia | 1 |  |  | 1 |
| America | 11 | 4 | 7 | 0 |
| Costa Rica | 3 |  | 3 |  |
| Dominican Republic | 3 | 3 |  |  |
| Peru | 2 | 1 | 1 |  |
| Brazil | 1 |  | 1 |  |
| Cuba | 1 |  | 1 |  |
| Nicaragua | 1 |  | 1 |  |
| India | 2 |  | 1 | 1 |
| Southeast Asia | 7 |  | 7 |  |
| Unknown | 6 | 3 | 3 |  |

Table S2. Treatment received by patients with malaria.

|  | Total | Admitted | Outpatients |
| --- | --- | --- | --- |
| IV artesunate | 7 | 7 | 0 |
| Completed with artemether/lumefantrine | 3 | 3 | – |
| Completed with atovacuone/proguanil | 3 | 3 | – |
| Atovaquone/proguanil | 82 | 61 | 21 |
| Atovacuone/proguanil + primaquine | 5 | – | 5 |
| Mefloquine | 5 | 4 | 1 |
| Artemether/lumefantrine | 4 | 4 | – |
| Artemether/lumefantrine + primaquine | 2 | 2 | – |
| No data available | 3 | 1 | 2 |

Table S3. Alternative diagnosis when malaria was excluded.

| Upper respiratory tract infections | 79 (35.9%) |
| --- | --- |
| Acute gastroenteritis or traveler’s diarrhea | 20 (9%) |
| Other tropical diseases | 9 (4%) |
| UTI’s | 9 (4%) |
| Acute tonsillitis | 9 (4%) |
| Tuberculosis | 8 (3.6%) |
| Community acquired pneumonia | 7 (3.2%) |
| HIV primo-infections | 2 (1%) |
| Others | 77 (35%) |
